# Mechanisms of action of monoclonal antibodies in oncology integrated in IMGT/mAb-DB

**DOI:** 10.3389/fimmu.2023.1129323

**Published:** 2023-05-05

**Authors:** Taciana Manso, Anjana Kushwaha, Nika Abdollahi, Patrice Duroux, Véronique Giudicelli, Sofia Kossida

**Affiliations:** IMGT, The International ImMunoGeneTics Information System, National Center for Scientific Research (CNRS), Institute of Human Genetics (IGH), University of Montpellier (UM), Montpellier, France

**Keywords:** IMGT, monoclonal antibodies, immune checkpoints, oncology, immunotherapy

## Abstract

**Background:**

Cancer cells activate different immune checkpoint (IC) pathways in order to evade immunosurveillance. Immunotherapies involving ICs either block or stimulate these pathways and enhance the efficiency of the immune system to recognize and attack cancer cells. In this way, the development of monoclonal antibodies (mAbs) targeting ICs has significant success in cancer treatment. Recently, a systematic description of the mechanisms of action (MOA) of the mAbs has been introduced in IMGT/mAb-DB, the IMGT® database dedicated to mAbs for therapeutic applications. The characterization of these antibodies provides a comprehensive understanding of how mAbs work in cancer.

**Methods:**

In depth biocuration taking advantage of the abundant literature data as well as amino acid sequence analyses from mAbs managed in IMGT/2Dstructure-DB, the IMGT® protein database, allowed to define a standardized and consistent description of the MOA of mAbs targeting immune checkpoints in cancer therapy.

**Results:**

A fine description and a standardized graphical representation of the MOA of selected mAbs are integrated within IMGT/mAb-DB highlighting two main mechanisms in cancer immunotherapy, either Blocking or Agonist. In both cases, the mAbs enhance cytotoxic T lymphocyte (CTL)-mediated anti-tumor immune response (Immunostimulant effect) against tumor cells. On the one hand, mAbs targeting co-inhibitory receptors may have a functional Fc region to increase anti-tumor activity by effector properties that deplete T_reg_ cells (Fc-effector function effect) or may have limited FcγR binding to prevent T_eff_ cells depletion and reduce adverse events. On the other hand, agonist mAbs targeting co-stimulatory receptors may bind to FcγRs, resulting in antibody crosslinking (FcγR crosslinking effect) and substantial agonism.

**Conclusion:**

In IMGT/mAb-DB, mAbs for cancer therapy are characterized by their chains, domains and sequence and by several therapeutic metadata, including their MOA. MOAs were recently included as a search criterion to query the database. IMGT® is continuing standardized work to describe the MOA of mAbs targeting additional immune checkpoints and novel molecules in cancer therapy, as well as expanding this study to other clinical domains.

## Graphical Abstract

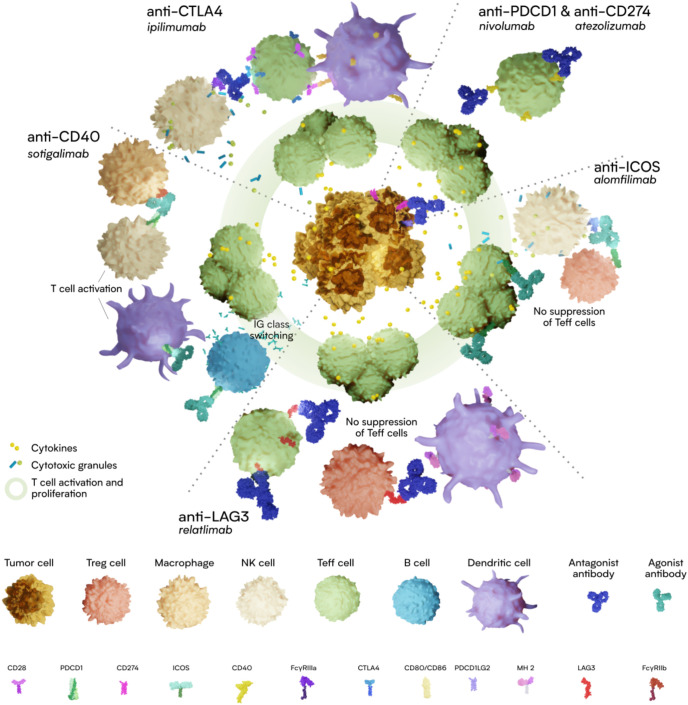

## Introduction

Cancer is the leading cause of mortality worldwide, accounting for an estimated 10 million deaths in 2020 (1). The immune system is intrinsically involved in the physiological fight against cancer, acting in the detection and elimination of the tumor. The capacity of malignant cells to express immunological checkpoint molecules on their surface is one strategy by which they avoid their destruction by the immune system. Immune checkpoints (ICs) consist of co-inhibitory and co-stimulatory proteins that activate pathways necessary for the balance of the immune functions and contribute to the regulation of the immune response. ICs in cancer allow tumors to evade and escape immune surveillance, in particular by inhibiting T cells activation (2, 3). Understanding the fundamental principles of cancer-immune system interactions allows a rational development of therapeutic strategies to activate and reinforce the immune system for cancer treatment.

**Figure 1:**
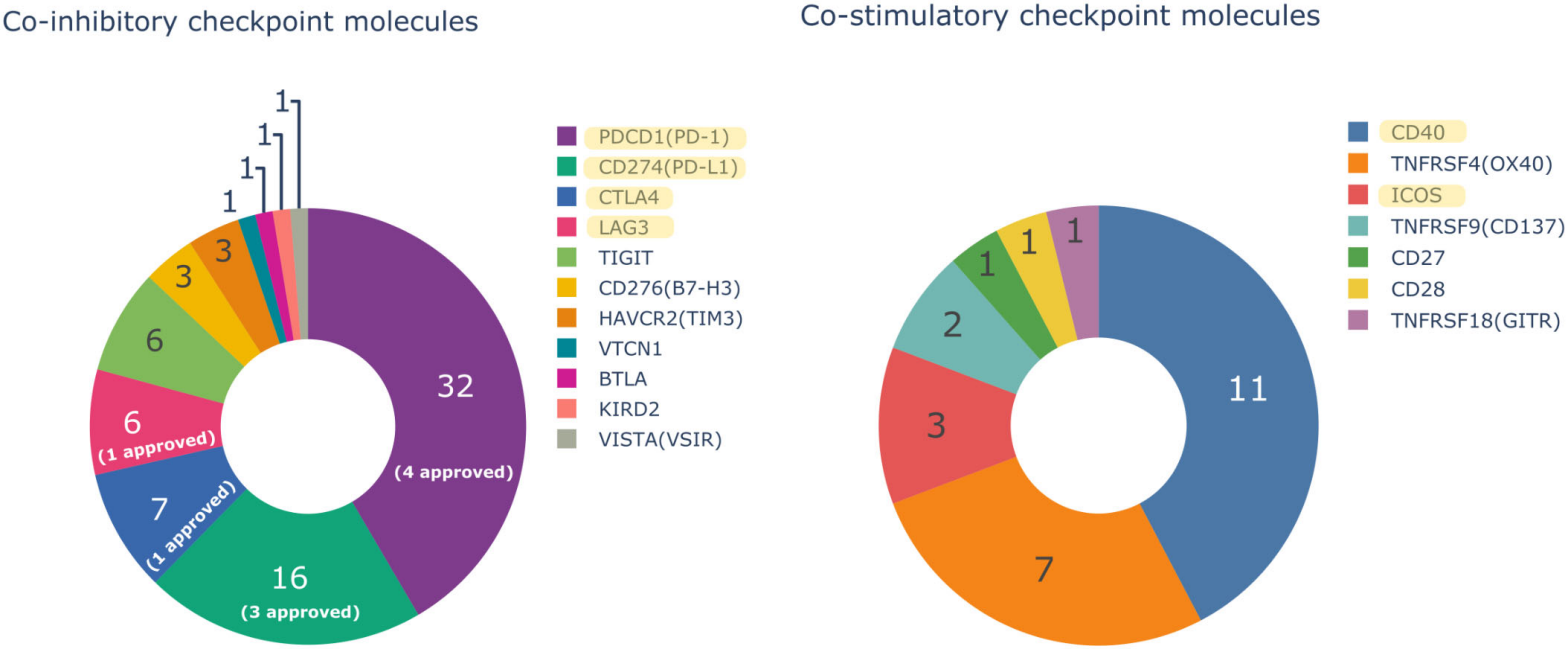
Number of monoclonal antibodies (mAbs) in oncology domain with an INN name assigned by the International Nonproprietary Names (INN) Program of the World Health Organization (WHO) targeting only one co-inhibitory or co-stimulatory immune checkpoint molecule and the number of Food and Drug Administration (FDA) and/or European Medicines Agency (EMA) approved mAbs. Bispecific mAbs are not considered in the Figure. Yellow highlighted targets are described in this study.

Monoclonal antibodies (mAbs) have mostly been employed in cancer immunotherapy throughout the previous few decades, showing an extremely promising potential in medicine (4). To date, the World Health Organization’s (WHO) International Nonproprietary Names (INN) Program has assigned INN names to about 1,000 mAbs (5), 530 of which are in the oncology domain. In 2011, the first immune checkpoint inhibitor (ICI) for cancer treatment, ipilimumab, was approved by FDA. Since then, more than 70,000 studies regarding “therapeutic monoclonal antibody” have been referenced by PubMed (reviews excluded). With $17 billion in sales, pembrolizumab (KEYTRUDA®), a mAb used to treat multiple cancers, was the world’s best-selling cancer drug in 2021. mAbs targeting emerging IC molecules to stimulate and improve T cell functions are now being developed in order to investigate potential co-signaling pathways that may enhance cancer therapy efficacy (**Figure 1**). Given the significance of having quick and easy access to reliable information about therapeutic mAbs for the scientific community, IMGT®, the international ImMunoGeneTics information system® (http://www.imgt.org) (6), since 2010, has offered to the community a unique and valuable resource concerning monoclonal antibodies with therapeutic application through its database, IMGT/mAb-DB. This database provides a one-of-a-kind resource on mAbs, fusion protein for immune application (FPIA), composite protein for clinical application (CPCA), related protein of the immune system (RPI), and T cell receptors (TR) with clinical indications. It includes INN names and definitions, sequence analysis, amino acid mutations and therapeutic metadata. The HGNC official names are also provided with a link to the target names (7).

As of January 2023, IMGT/mAb-DB contains 1,342 entries: 1,167 IG, 65 CPCA, 61 RPI, 44 FPIA and 5 TR from several clinical domains. In the field of oncology, IMGT/mAb-DB has 530 mAbs, with assigned INN names, in different clinical trials. Among these mAbs, 54 have been approved by the U.S. Food and Drug Administration (FDA) and/or European Medicines Agency (EMA). Regarding immune checkpoint molecules, IMGT/mAb-DB includes 148 mAbs targeting an IC with an assigned INN name, of which nine have been approved by FDA and/or EMA. Sources of data managed in IMGT/mAb-DB are extracted from INN/WHO Proposed and Recommended lists. These lists provide, for each mAb, an INN definition based on the amino acid (AA) sequences and biochemical properties of the mAb chains, domains and regions. The AA sequences are analyzed and stored in IMGT/2Dstructure-DB (8), which is linked to IMGT/mAb-DB.

Since 2020, IMGT^®^ progressively extends the characterization of therapeutic antibodies in oncology with the description of their mechanisms of action (MOA) on different targets, mainly immune checkpoints. ICs play an essential role in the regulation of T cells, that can recognize and attack malignant cells. A very thorough understanding of the anti-tumor immune response is required for efficient and safe ICI therapy. Thus, six major ICs for cancer immunotherapy were analyzed in this work, namely CTLA4, PDCD1, CD274, ICOS, LAG3, and CD40. The aim is to provide a clear understanding of how monoclonal antibodies act in cancer, with valuable insights towards targeted and personalized therapies with effectiveness of mAbs in human diseases. Lastly, the MOA description resulted in the creation of two new concepts in the IMGT-ONTOLOGY (9), ‘Mechanism of action’ and ‘Effect’ as well as the associated vocabulary for a uniform definition of MOA. These concepts, integrated in the web interface, correspond to new criteria to query the database.

## Methods

Based on the co-stimulatory and co-inhibitory immune checkpoints, we carried out literature searches concerning mAbs targeting the six major ICs for cancer immunotherapy. These targets can be selected in the “Specificity target name” field in IMGT/mAb-DB query page. The mAbs retrieved, were studied and their MOA described in this work. The antibodies are only referred to by their INN name. Bispecific antibodies, fusion proteins and combination therapies are not dealt with in this study.

Scientific articles concerning the target and the mAb were investigated to provide a synthesis of each mAb’s MOA, following two main approaches: (i) for well-studied targets and antibodies with their defined mechanisms of action, data from the literature were extracted to standardize the explanation of the MOA (ii) for new targets and antibodies that do not have a well-defined MOA, data from the literature were used to describe the target’s function in cancer, in order to suggest a mechanism of action of the antibodies (in such cases, the notification “proposed by IMGT” is added in the MOA description). In both approaches, a synthesis was performed for the mAb’s function in cancer immunosurveillance evasion and a description of the MOA was provided to tie together all the material provided. Subsequently, using IMGT/2Dstructure-DB and IMGT/DomainGapAlign tool (10), the AA sequences of each mAb was examined to look for mutations introduced in the Fc (Fragment crystallizable) region of the antibodies, in order to identify modifications in FcγRs (Fc-gamma receptors) binding and effector properties of the MOA.

The MOA of each antibody was illustrated through a standardized schematic representation utilizing the AFFINITY Designer tool (Serif, RRID : SCR_016952). To allow a good representation of the protein interactions, the real size of the proteins in relation to the size of the cell surface was not respected. It is worth noting that the real size of an IG is about 10 nm, a B cell diameter is about 7 µm, and a T cell diameter is about 6 µm, both cells without stimulation (11, 12). In addition to the schema, a terminology was established by IMGT®, based on the NCI Thesaurus terminology (https://ncit.nci.nih.gov/ncitbrowser/), to provide keywords that describe the main mechanism of action of each mAb and its specific effects (**Table 1**). This terminology is constantly enriched as new mAbs are studied from different clinical domains.

**Table 1:** Terminology established by IMGT® to describe the mechanisms of action of monoclonal antibodies, studied in this work, in the oncology domain.

Terms	Definition
Mechanism of action	
Blocking	A molecule that binds to a receptor or a ligand and inhibits its activity
Agonist	A molecule that activates a receptor and induces its biological response
Effect	
Immunostimulant	A molecule that stimulates the immune system activity
Fc-effector function	A molecule with effector function against a target cell such as cellular cytotoxicity (ADCC), cellular phagocytosis (ADCP) and/or complement-dependent cytotoxicity (CDC)
FcyR crosslinking	A molecule that binds to FcγRIIb to trigger antibody crosslinking (clustering) and strong agonism

A standardized description was created for each mAb’s MOA. It includes the HGNC gene name for the target and its abbreviation, keywords to describe the mechanism and the expected immune response, and, if applicable, descriptions of mutations in the Fc region and their effects on the Fc-effector function. A query on IMGT/mAb-DB interface (https://www.imgt.org/mAb-DB/), using the established keywords, allows access to the description of a given MOA and its schematic representation for the set of mAbs that use this same MOA.

## Results

### Monoclonal antibodies targeting CTLA4

Cytotoxic T cell lymphocyte-associated protein 4 (CTLA4) is a transmembrane protein member of the CD28 family receptor expressed by T cells, constitutively by CD4+ CD25+ regulatory T cells (T_reg_) and only following activation by cytotoxic CD8+ effector T cells (T_eff_) (13). In general, CTLA4 counteracts the activity of CD28, a co-stimulatory molecule expressed by T cells. Following antigen recognition, CD28 binds to CD80 (also known as B7-1) and CD86 (also known as B7-2), expressed by activated antigen-presenting cells (APCs), and transmits a co-stimulatory signal for T cell activation and proliferation (14). CTLA4 is placed in intracellular vesicles and directed on the T cell surface only after T cell receptor (TR) activation (15), where it binds to CD80/CD86 with greater affinity than that of CD28, transmitting co-inhibitory signals to control T_eff_ cell activation (14, 16) and preventing the potential damage by an excessive inflammatory response (14–16).

**Figure 2:**
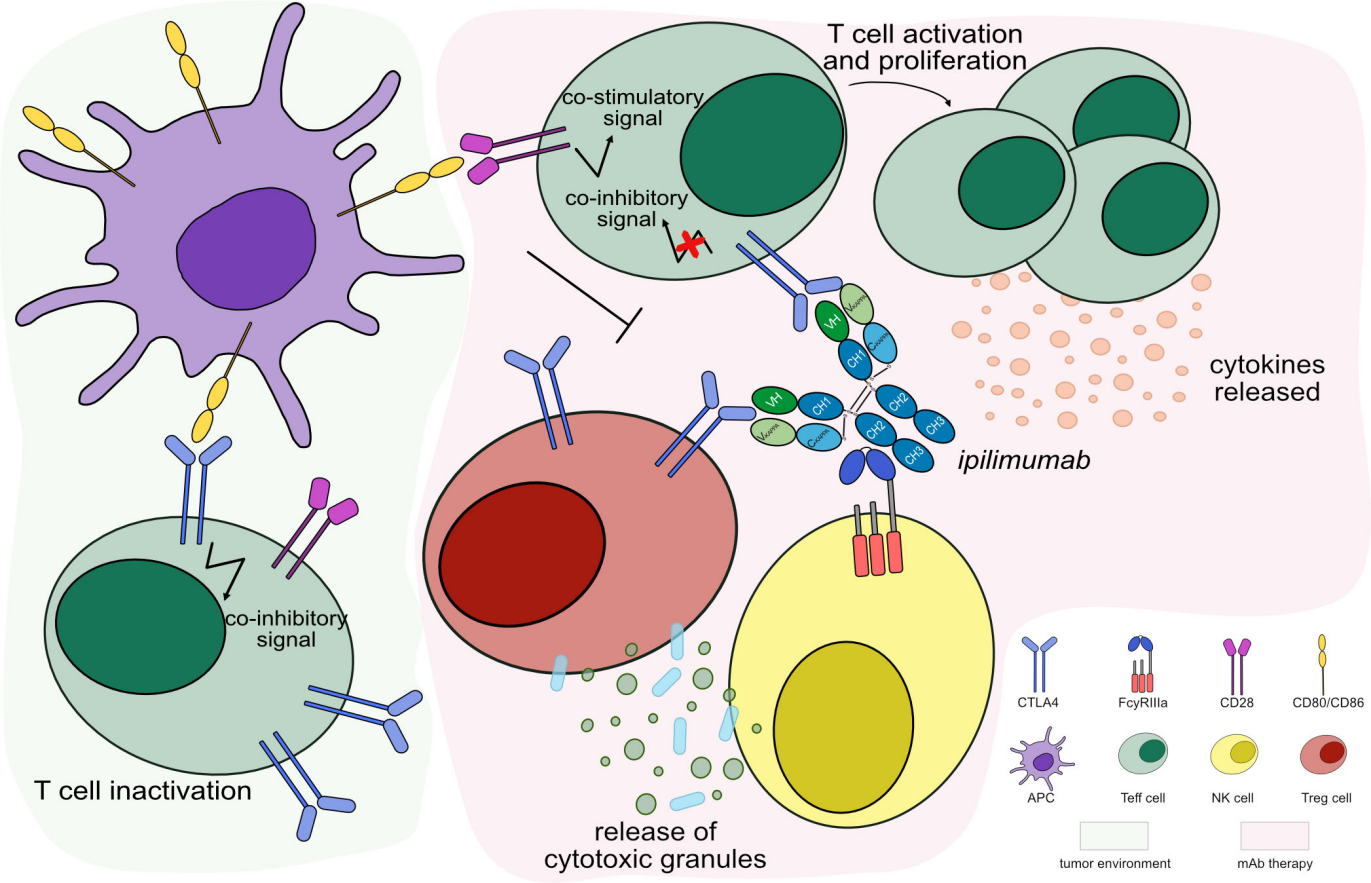
Mechanism of action of blocking mAb targeting CTLA4. Light green background shows CTLA4 binding in tumor microenvironment: CTLA4, highly expressed by activated T cells binds to its ligands, CD80/CD86 expressed by APC, with higher affinity than CD28, leading to T cell inactivation. Light pink background illustrates the mAb therapy: the antibody blocks CTLA4 from binding to its ligands and promotes CD80/CD86 binding to CD28 receptor. This restores the stimulatory CD28 pathway to activate T_eff_ cells, which enhance cytotoxic activity against tumor cells. The IgG1-Fc region of *ipilimumab* binds to FcγRIIIa and induces ADCC to increase the anti-tumor activity by T_reg_ cells depletion. Mechanism of action: Blocking. Effect: Immunostimulant, Fc-effector function. (mAb ID 180).

IMGT/mAb-DB lists eight mAbs targeting CTLA4 for cancer immunotherapy. Ipilimumab (YERVOY®, IMGT/mAb-DB ID: mAbID 180) blocks the binding of CTLA4 with its ligands, then inhibits CTLA4-mediated downregulation of T cells and promotes the interaction of CD80/CD86 with CD28. This interaction stimulates the immune response by increasing T cell expansion and by enhancing the cytotoxic T lymphocyte (CTL)-mediated anti-tumor immune response (17). By its effector properties, the IgG1-Fc region of ipilimumab induces antibody-dependent cellular cytotoxicity (ADCC) and complement-dependent cytotoxicity (CDC) for enhanced anti-tumor efficacy by reducing T_reg_ cells (17, 18) (**Figure 2**). Thus, ipilimumab’s MOA is ‘Blocking - Immunostimulant, Fc-effector function’ (**Table 2**). In contrast, tremelimumab (mAbID 248) differs from ipilimumab in its Fc region and its ability to engage FcγRs. The IgG2-Fc region of tremelimumab shows reduced affinity to various FcγRs (19) and presents minimal ADCC activity against cancer cells (20). Therefore, tremelimumab’s MOA is ‘Blocking - Immunostimulant’ without Fc-effector function to deplete T_reg_ cells.

**Table 2:** Blocking anti-CTLA4 mAbs present in IMGT/mAb-DB and their mechanisms of action (MOA).

INN mAbs	Isotype	IMGT variant (Fc-enhanced)	IMGT MOA	Clinical trial status
botensilimab	IgG1	G1v8	**Blocking** Immunostimulant, Fc-effector function	Phase II (NCT05529316)
ipilimumab	IgG1	–		Phase M (first approval in 2011)
porustobart	IgG1	G1v7		Phase I (NCT04135261)
quavonlimab*	IgG1	–		Phase III (NCT04736706)
tuvonralimab*	IgG1	–		Phase I (NCT05171790)
zalifrelimab	IgG1	–		Phase I/II (NCT02694822)
tremelimumab	IgG2	–	**Blocking** Immunostimulant	Phase M (first approval in 2022)
nurulimab**	IgG1	–	–	Phase I (NCT03472027)

*Monoclonal antibodies with a MOA suggested by IMGT owing to a lack of scholarly papers giving proof of their pre-clinical effects. Their MOA may evolve as new data emerge. IMGT suggestion is based on i) the function of the mAb target in the cancerous environment and ii) the analysis of their Fc region, when possible.

** No information from the literature to describe the MOA.

It is worth noting that antibodies with high affinity to FcγRs may increase the anti-tumor activity (18). Thus, enhancing FcγR binding by modifying the Fc region provided a generation of engineered anti-CTLA4 antibodies with increased anti-tumor activity by T_reg_ cells depletion (18). These engineered antibodies act in the same way as ipilimumab, blocking CTLA4 to stimulate CTL activity, and enhancing Fc-effector function against T_reg_ cells (**Table 2**). Botensilimab (mAbID 1123), an anti-CTLA4 antibody with enhanced Fc-effector function, has been engineered to improve FcγRIIIa affinity while decreasing FcγRIIb binding and boosting effector functions such as ADCC (21). **Table S1** and IMGT Biotechnology page (https://www.imgt.org/IMGTbiotechnology/ > Antibody glycosylation and effector properties > IMGT engineered variant nomenclature: IGHG variants) describe in detail and in a standardized format all engineered antibodies Fc variants (22) provided in this study.

### Monoclonal antibodies targeting PDCD1/CD274

Programmed death receptor 1 (PDCD1, PD-1) is a member of the CD28/CTLA4 family receptors that downregulates T cell activation, proliferation, and cytotoxic activity in peripheral tissues during inflammatory responses. PDCD1 is expressed by activated T cells, B cells as well as natural killer (NK) cells and upregulated on T cells after persistent antigen exposure, preventing autoimmunity (23). The expression of its ligands, programmed death ligand 1 (CD274, PD-L1) and programmed death ligand 2 (PDCD1LG2, PD-L2, CD273), is induced by inflammatory cytokines released after TR activation, on tumor cells (23, 24). Thus, the binding of PDCD1 to its ligands downregulates T_eff_ cell activity and promotes tumor escape (23, 25). PDCD1 and other co-inhibitory molecules, such as LAG3, could lead to T cell exhaustion. Exhausted T_eff_ cells lose several functions such as interleukin 2 (IL2) production, proliferative capacity, and cytotoxicity (26).

Monoclonal antibodies targeting the PDCD1 axis include 1) molecules directed to PDCD1, blocking receptor interaction with both ligands, as well as 2) antibodies against CD274, blocking ligand interaction with PDCD1. Both therapeutic approaches enhance immune mediated anti-tumor responses in several cancer types, including melanoma and bladder cancer (27, 28).

**Figure 3:**
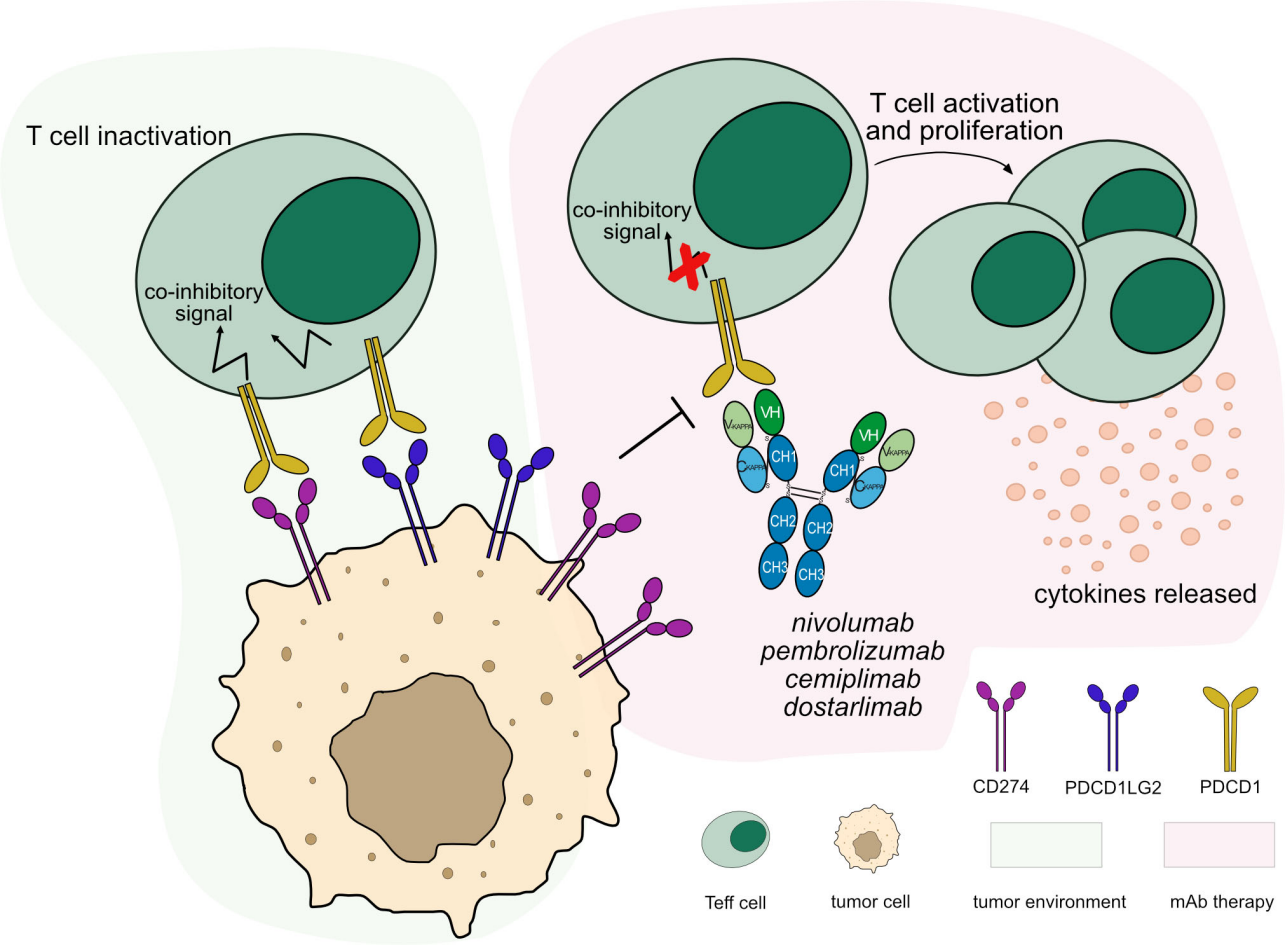
Mechanism of action of blocking mAbs targeting PDCD1. Light green background shows PDCD1 binding in tumor microenvironment: PDCD1 (PD-1) expressed by T cells binds to its ligands, CD274 (PD-L1) and PDCD1LG2 (PD-L2) expressed by tumor cells leading to T cell inactivation. Light pink background illustrates the mAb therapy: the antibodies block PDCD1 from binding to its ligands. This restores the activation and proliferation of T_eff_ cells, which enhance cytotoxic activity against tumor cells. These antibodies are hinge-stabilized IgG4 (IMGT variant G4v5 h P10) with limited FcγR-binding to prevent T_eff_ cells depletion. Mechanism of action: Blocking. Effect: Immunostimulant. (mAb IDs 424, 472, 846, 849).

Currently, IMGT/mAb-DB includes 32 anti-PDCD1 mAbs for cancer immunotherapy. To date, four anti-PDCD1 mAbs have been approved by FDA and/or EMA for different cancer types, nivolumab (OPDIVO®, mAbID 424), pembrolizumab (KEYTRUDA®, mAbID 472), dostarlimab (JEMPERLI, mAbID 849) and cemiplimab (LIBTAYO®, mAbID 846). These antibodies block the PDCD1 receptor from binding to both ligands, CD274 and PDCD1LG2. This process reverses T cell inactivation and restores immune function through the activation of CTL against tumor cells (29, 30) (**Figure 3**). Therefore, the antibodies’ MOA is ‘Blocking - Immunostimulant’ (**Table S2**).

Most of the anti-PDCD1 mAbs are IgG4 subclass, which presents low affinity to FcγRs (31) and little ability to mediate cellular cytotoxic effector functions against T_eff_ cells. Therapeutic IgG4 mAbs are designed to stabilize and prevent half-IG molecules by a single mutation (IMGT variant G4v5 h P10) introduced in the hinge region (32, 33). Attempts have been made to introduce mutations in IgG4 antibodies to completely abolish FcγR binding and avoid any cytotoxicity, an example being tislelizumab (mAbID 757) (34, 35). The usage of IgG1 subclass with abolished FcγR binding was explored, as in the case of penpulimab (mAbID 1093) (36) and some other additional mAbs (**Table S2**). For more details about IMGT engineered variants, see **Table S1**.

As mentioned above, there exist antibodies which bind to CD274, blocking the ligand-receptor interaction. IMGT/mAb-DB includes 17 anti-CD274 mAbs, of which three have been approved by FDA and/or EMA, atezolizumab (TECENTRIQ®, mAbID 526), durvalumab (IMFINZI™, mAbID 528) and avelumab (BAVENCIO®, mAbID 512).

**Figure 4:**
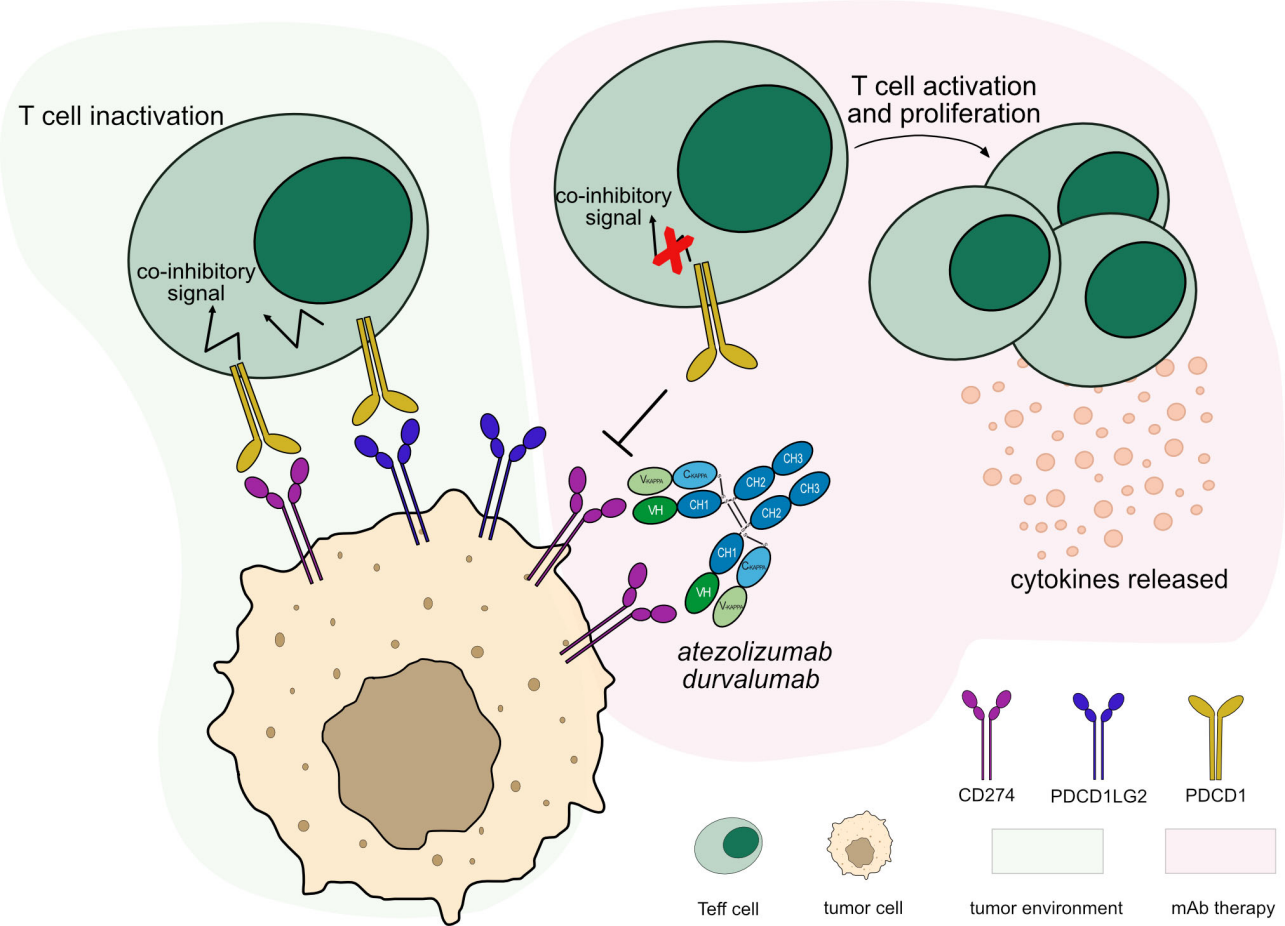
Mechanism of action of blocking mAbs targeting CD274. Light green background shows CD274 binding in tumor microenvironment: PDCD1 (PD-1) expressed by T cells binds to CD274 (PD-L1) expressed by tumor cells leading to T cell inactivation. Light pink background illustrates the mAb therapy: the antibodies block CD274 from binding to PDCD1 expressed by activated T cells, leaving the PDCD1 pathway intact through its second ligand, PDCD1LG2 (PD-L2), to maintain immune tolerance. This blockade restores the activation and proliferation of T_eff_ cells which enhance cytotoxic activity against tumor cells. The IgG1-Fc region of the antibodies have been engineered to minimize FcγRs binding and ADCC/CDC activity, preventing T_eff_ cells depletion. Mechanism of action: Blocking. Effect: Immunostimulant. (mAb IDs 526, 528).

Blockade caused by anti-CD274 mAb binding restores T_eff_ cell activation and enhances cytotoxic immune response against tumor cells. The anti-CD274 antibody does not block the PDCD1/PDCD1LG2 pathway, allowing inhibitory signals to maintain immune tolerance (37) (**Figure 4**). Several anti-CD274 antibodies have been developed to avoid Fc effector functions by mutations in the IgG1-Fc region or by using IgG4 subclass (**Table S1**), to prevent T_eff_ cells depletion, such as atezolizumab (38) and durvalumab (39). Their main MOA is ‘Blocking - Immunostimulant’ (**Table 3**).

**Table 3:** Blocking anti-CD274 mAbs present in IMGT/mAb-DB and their mechanisms of action (MOA).

INN mAbs	Isotype	IMGT variant (Fc-silenced)	IMGT MOA	Clinical trial status
adebrelimab*	IgG4	G4v4	**Blocking** Immunostimulant	Phase III (NCT05496166)
atezolizumab	IgG1	G1v29		Phase M (first approval in 2016)
betifisolimab	IgG1	G1v29		Phase I (NCT03463473)
durvalumab	IgG1	G1v39		Phase M (first approval in 2017)
envafolimab	IgG1	G1v71		Phase II (NCT04480502)
garivulimab	IgG1	G1v4		Phase I/II (NCT03379259)
pacmilimab	IgG4	–		Phase II (NCT04596150)
sugemalimab	IgG4	–		Phase II/III (NCT05623267)
avelumab	IgG1	–	**Blocking** Immunostimulant, Fc-effector function	Phase M (first approval in 2017)
cosibelimab*	IgG1	–		Phase III (NCT04786964)
danburstotug*	IgG1	–		Phase II (NCT03999658)
socazolimab*	IgG1	–		Phase III(NCT04878016, NCT04359550)
lesabelimab**	IgG1	–	–	Phase II (NCT04718584)
manelimab**	IgG1	–	–	Phase I
opucolimab**	IgG1	–	–	Phase I (NCT03588650)
sudubrilimab**	IgG1	–	–	–
tagitanlimab**	IgG1	–	–	Phase III (NCT05294172)

*Monoclonal antibodies with a MOA suggested by IMGT owing to a lack of scholarly papers giving proof of their pre-clinical effects. Their MOA may evolve as new data emerge. IMGT® suggestion is based on i) the function of the mAb target in the cancerous environment and ii) the analysis of their Fc region, when possible.

** No information from the literature to describe the MOA.

Unlike other anti-CD274 mAbs which are designed to eliminate any ADCC/CDC activity as a precaution of off-tumor cytotoxicity, the IgG1-Fc region of avelumab binds to FcγRs on NK cells and directly mediates cellular cytotoxicity against tumor cells (40). According to its properties, its MOA is classified as ‘Blocking - Immunostimulant, Fc-effector function’.

### Monoclonal antibodies targeting ICOS

Inducible T cell co-stimulator (ICOS) belongs to the CD28/CTLA4 family of receptors that stimulates immune response and homeostasis (41). ICOS is a homodimeric transmembrane protein expressed upon TR engagement and CD28 signaling on activated T cells (42, 43). The binding of ICOS with its ligand, ICOSLG (CD275) expressed by APCs (44), promotes proliferation and differentiation of T_eff_ and T_reg_ cells (45).

ICOS co-stimulation promotes, on the one hand, anti-tumor CTL activation which produces inflammatory cytokines (43, 45) and, on the other hand, T_reg_ cells proliferation which enhances tumor activity (46). This dual controversy effect of ICOS/ICOSLG interaction represents an attractive target to explore for mAbs engineering. It is worth noting that ICOS expression varies depending on T cell subtypes and on their localization; intratumoral T_reg_ cells exhibit higher ICOS expression than T_eff_ cells (43). This differential expression plays an important role in the MOA of the engineered mAbs as presented below.

mAbs targeting ICOS have been developed, however none of them have reached the clinic yet. IMGT/mAb-DB lists three mAbs targeting ICOS, with an assigned INN name, for cancer immunotherapy. A first approach was to develop agonist mAbs targeting ICOS that activate this signaling and exert anti-tumor activity by co-stimulating low ICOS+ T_eff_ cells, thereby promoting activation and expansion of T_eff_ cells, which in turn increase cytotoxic activity against tumor cells.

**Figure 5:**
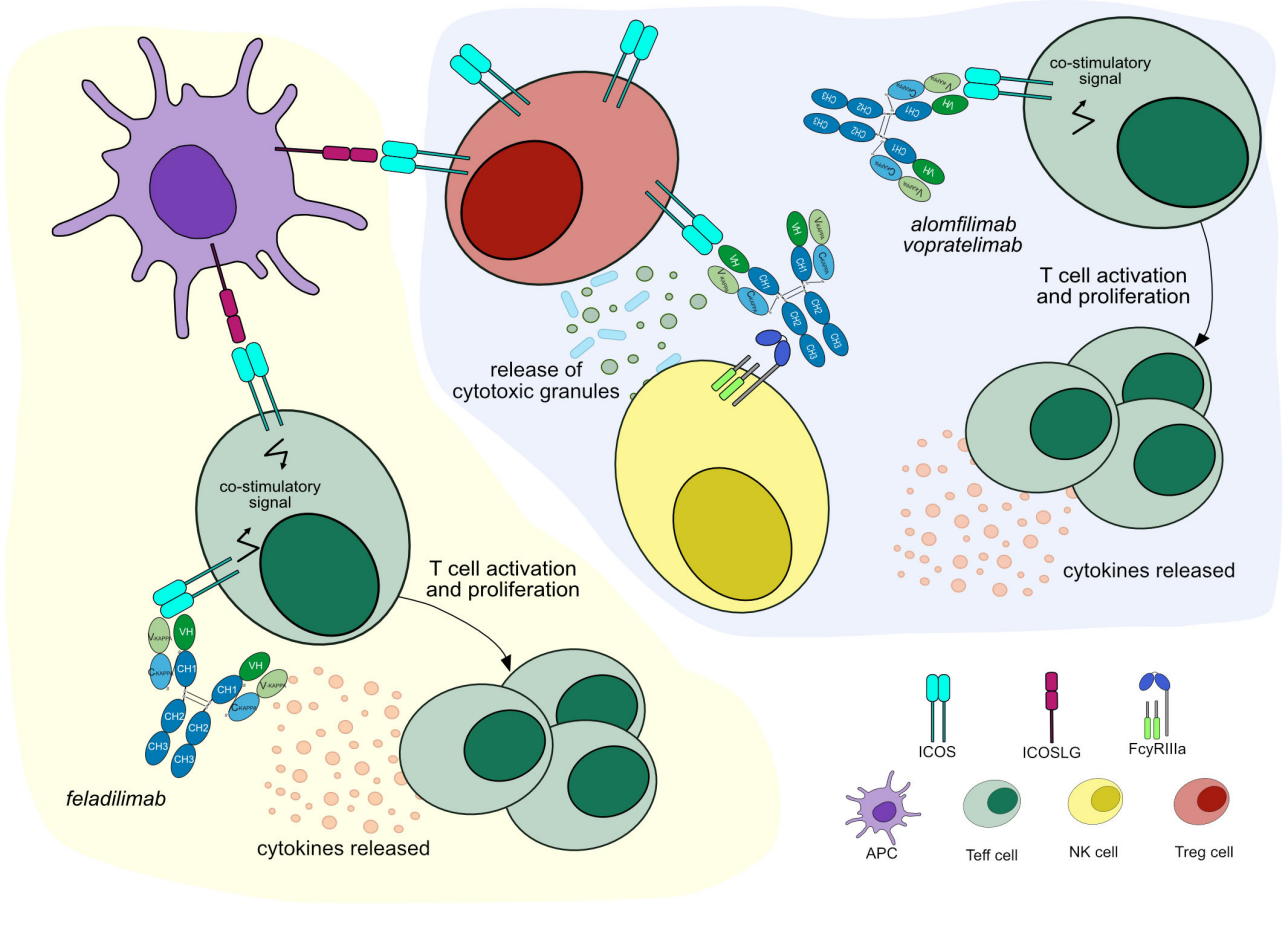
Mechanisms of action of agonist mAbs targeting ICOS. Light yellow background shows mAb therapy by *feladilimab*: the antibody stimulates ICOS signaling to activate T_eff_ cells, which enhance cytotoxic activity against tumor cells. *Feladilimab* has one mutation in the CH2 domain (IMGT variant G4v3 CH2 E1.2) to reduce ADCC/CDC, preventing T_eff_ cells depletion. Mechanism of action: Agonist. Effect: Immunostimulant. (mAb ID 1010). Light purple background shows mAb therapy by *alomfilimab* and *vopratelimab*: the antibodies stimulate ICOS signaling to activate low ICOS+ T_eff_ cells and promote cytotoxic anti-tumor immune response. Both antibodies bind to FcγRIIIa and induce ADCC to deplete high ICOS+ T_reg_ cells. Mechanism of action: Agonist. Effect: Immunostimulant, Fc-effector function. (mAb ID 801).

Feladilimab (mAbID 1010), a humanized IgG4-kappa mAb, acts as an agonist of ICOS, activating T_eff_ cells, which mediate a cytotoxic anti-tumor immune response (47). Thus, feladilimab’s main MOA is ‘Agonist - Immunostimulant’ (**Figure 5** and **Table 4**). Feladilimab has an engineered Fc region (IMGT variant G4v3 CH2 E1.2) which reduces C1q and FcγR binding (**Table S1**) and avoids ICOS+ T_eff_ cells depletion by ADCC activity. However, its agonist activity is not sufficient to induce anti-tumor cytotoxicity (41), therefore feladilimab was discontinued in phase II trials due to cancer progression.

**Table 4:** Agonist mAbs targeting ICOS present in IMGT/mAb-DB and its mechanisms of action (MOA).

INN mAbs	Isotype	IMGT variant (Fc-silenced)	T_eff_ activation	T_reg_ depletion	IMGT MOA	Clinical trial status
feladilimab	IgG4	G4v3	✔	–	**Agonist** Immunostimulant	Discontinued
alomfilimab	IgG1	–	✔	✔	**Agonist** Immunostimulant, Fc-effector function	Phase I/II (NCT03829501)
vopratelimab	IgG1	–	✔	✔		Phase III (NCT03989362)

An alternative approach aims to deplete intratumoral high ICOS+ T_reg_ cells by Fc effector functions (48, 49). Equivalently to feladilimab, alomfilimab (mAbID 1120) and vopratelimab (mAbID 801) activate and induce the proliferation of low ICOS+ T_eff_ cells, enhancing CTL-mediated anti-tumor immune response. Nevertheless, in addition to feladilimab’s MOA, these antibodies have an effect of ‘Fc-effector function’ in cancer treatment and preferentially deplete high ICOS+ T_reg_ cells by Fc effector function (50, 51) (**Figure 5** and **Table 4**). The T_reg_ cells depletion ability is triggered when high levels of antigen are found on the surface of the target cell, the case of T_reg_ cells, promoting signaling through FcγR clusters in immune effector cells and strong ADCC against target cells (52).

### Monoclonal antibodies targeting LAG3

Lymphocyte activation gene-3 protein (LAG3, also known as CD223) is a member of IG superfamily expressed by T cells upon antigen stimulation, B cells and NK cells (53, 54). The LAG3 signaling pathway plays a critical role inhibiting T cell activation and proliferation while stimulating differentiation into T_reg_ cells which leads to immunosuppression (55). The LAG3 inhibitory function is closely correlated with the LAG3 expression levels on the T cell surface (56). Thereby, a constant antigen stimulation of the T cells in the tumor microenvironment (TME) leads to a high expression of LAG3 and of other co-inhibitory receptors on T cells, such as PDCD1, promoting immune escape in tumors and exhaustion of T cells that lose their effector functions (57, 58).

LAG3 binds the major histocompatibility class II (MH2) and several other ligands, including galectin-3 (LGALS3, Gal-3), liver-secreted fibrinogen-like protein 1 (FGL1), and C-type lectin domain family 4 member G (CLEC4G, LSECtin). Binding to MH2, LAG3 inhibits CD4+ T cells activation (59). LGALS3 is secreted by many tumor cells and associated with neoplastic transformation. LAG3-LGALS3 interaction inhibits T cell responses promoting cancer progression (60). FGL1 plays a role in proliferation and metabolism and can be expressed by tumor cells. Its binding to LAG3 results in T cell depletion, mechanisms of immune evasion of the cancer and resistance to anti-PDCD1/CD274 therapy (61). Ultimately, CLEC4G is a type II transmembrane protein expressed by APCs and certain types of cancer. It interacts with LAG3 and prevents T_eff_ cell responses in melanoma cells (62).

**Figure 6:**
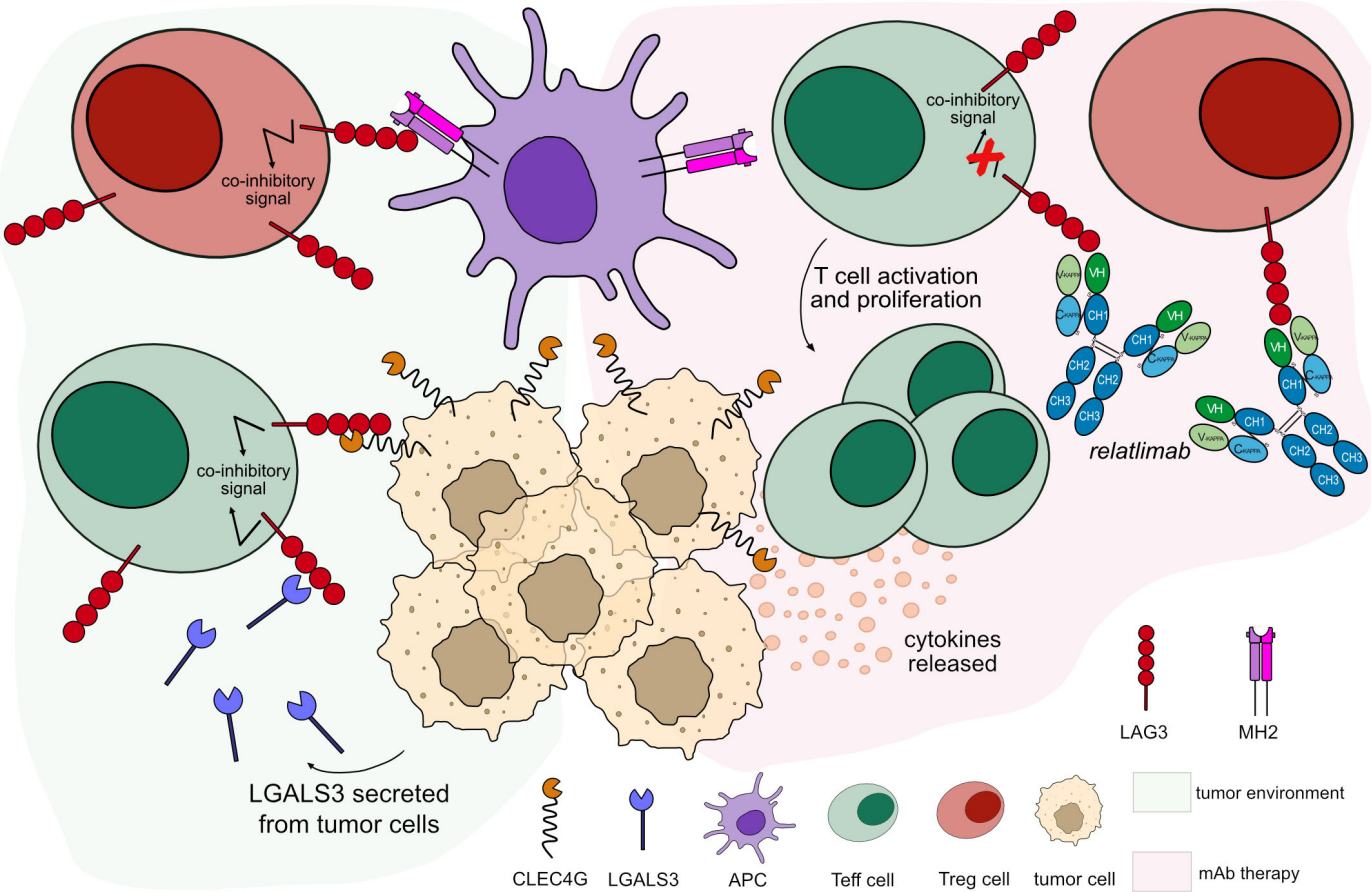
Mechanism of action of blocking mAb targeting LAG3. Light green background shows LAG3 binding in tumor microenvironment: LAG3 expressed by T cells binds to its ligands expressed by tumor cells and APCs, leading to T cell inactivation. Light pink background illustrates the mAb therapy: the antibody blocks LAG3 from binding to its ligands. This blockade restores the activation and proliferation of T_eff_ cells, which enhance cytotoxic activity immune response. *Relatlimab* is hinge-stabilized IgG4 antibody (IMGT variant G4v5 h P10) with limited FcγR-binding to prevent T_eff_ cells depletion. Mechanism of action: Blocking. Effect: Immunostimulant. (mAb ID 781).

There are several anti-LAG3 mAbs in different clinical trials; most of them are hinge-stabilized IgG4 antibodies (IMGT variant G4v5 h P10) to limit the FcγR binding. IMGT/mAb-DB includes seven anti-LAG3 antibodies, with an assigned INN name. Relatlimab (OPDUALAG™, mAbID 781), recently approved by FDA in 2022, binds to LAG3 overexpressed by T cells in the TME and blocks its binding to its ligands, activating exhausted T cells and enhancing the CTL-mediated immune response against tumor cells (63) (**Figure 6**). In combination with nivolumab, relatlimab demonstrated a progression-free survival increased when compared to nivolumab alone (64, 65). The main MOA of relatlimab is ‘Blocking – Immunostimulant’ (**Table 5**).

**Table 5:** Blocking anti-LGA3 mAbs present in IMGT/mAb-DB and their mechanisms of action (MOA).

INN mAbs	Isotype	IMGT variant (Fc-silenced)	IMGT MOA	Clinical trial status
encelimab	IgG4	–	**Blocking** Immunostimulant	Phase I (NCT02817633, NCT03250832)
favezelimab	IgG4	–		Phase III (NCT05600309, NCT05508867)
fianlimab	IgG4	G4v38		phase III (NCT05352672)
ieramilimab*	IgG4	–		Phase II completed (NCT03365791)
miptenalimab	IgG4	–		Phase II (NCT03697304)
relatlimab	IgG4	–		Phase M (first approval in 2022)
tuparstobart*	IgG1	G1v29		phase II (NCT04586244, NCT04463771)

*Monoclonal antibodies with a MOA suggested by IMGT owing to a lack of scholarly papers giving proof of their pre-clinical effects. Their MOA may evolve as new data emerge. IMGT suggestion is based on i) the function of the mAb target in the cancerous environment and ii) the analysis of their Fc region, when possible.

Assays have been made to introduce mutations in IgG4 antibodies in order to eliminate FcγR binding, such as fianlimab (mAbID 950) (66). The usage of IgG1 subclass with abolished FcγR binding was also explored, as in the case of tuparstobart (mAbID 1371) (67) (**Tables 5**, **S1**). This approach allows to design blocking mAbs totally silent to avoid cytotoxic activity against the target immune cells.

### Monoclonal antibodies targeting CD40

CD40 (also known as TNFRSF5) is a co-stimulatory immunoreceptor member of the TNFR superfamily which is expressed by APCs and B cells. CD40 is involved in T cell activation by dendritic cells (DCs) and in antibody class-switching in B cells (68, 69). Upon activation by its ligand, CD40LG (CD154) expressed by activated T cells, CD40 promotes APC activation and differentiation. It also promotes a bi-directional signaling between T cells and APCs, that amplifies a stimulatory immune response, increasing T cell expansion and enhancing CTL activity in the TME (70). In cancer immunotherapy, through the binding with agonist mAbs, CD40 can stimulate T cells and APCs to attack tumor cells (71, 72). In contrast, CD40 is highly expressed by B cell malignancies including non-Hodgkin’s lymphoma (NHL), chronic lymphocytic leukemia (CLL) and myeloma, and in that case, it promotes proliferation and inhibits the apoptosis of malignant B cells (73).

Monoclonal antibody therapy targeting CD40 acts via multiple mechanisms to stimulate anti-tumor immunity in a wide range of lymphoid and solid malignancies (74). Several mAbs targeting CD40 have been developed in the oncology domain. Twelve mAbs targeting CD40, with an assigned INN name, are integrated in IMGT/mAb-DB. However, none of them has reached the clinic yet. Agonist mAbs stimulate CD40 signaling of different pathways depending on the target cell type. However, in all cases, they promote cell activation and proliferation of several immune cells that contribute to anti-tumor activity. An ideal agonist mAb can lead to CD40 crosslinking to promote greatest agonist and anti-tumor activities with minimal adverse events (75).

The anti-CD40 mAb, selicrelumab (mAbID 723), has a strong agonist activity, since it does not block CD40LG binding site, and reduces CDC and ADCC activities against the target cell, thanks to its belonging to the IgG2 subclass (76, 77). In addition, the IgG2 subclass aids the agonist effect due to its lack of flexibility in the hinge region, which may trigger CD40 aggregation without FcγR engagement (78). The MOA of selicrelumab is ‘Agonist - Immunostimulant’.

Most agonist mAbs targeting CD40 are of the IgG1 subclass with different levels of affinity to CD40 and depend on crosslinking with FcγRs in order to facilitate CD40 aggregation for APCs activation (79, 80) as well as an ADCC activity against tumor cells (81, 82) (**Table 6**). Engineered mAbs with enhanced binding to FcγRIIIa and ADCC activity have been developed to increase CD40 crosslinking (**Table S1**). However, an increased affinity for FcγR could enhance not only anti-tumor activity but also adverse events, such as thrombocytopenia and transaminitis (80).

**Table 6:** Agonist mAbs targeting CD40 present in IMGT/mAb-DB and their mechanisms of action (MOA).

INN mAbs	Isotype	IMGT variant (Fc-enhanced)	IMGT MOA	Clinical trial status
cifurtilimab	IgG1	–	**Agonist** Immunostimulant, Fc-effector function	Phase II (NCT04993677)
dacetuzumab	IgG1	–		Discontinued
mitazalimab	IgG1	–		Phase I/II (NCT04888312)
dalnicastobart	IgG1	G1v47	**Agonist** Immunostimulant, FcγR crosslinking	Phase I (NCT05075993)
giloralimab	IgG1	G1v72		Phase II (NCT04807972)

**Figure 7:**
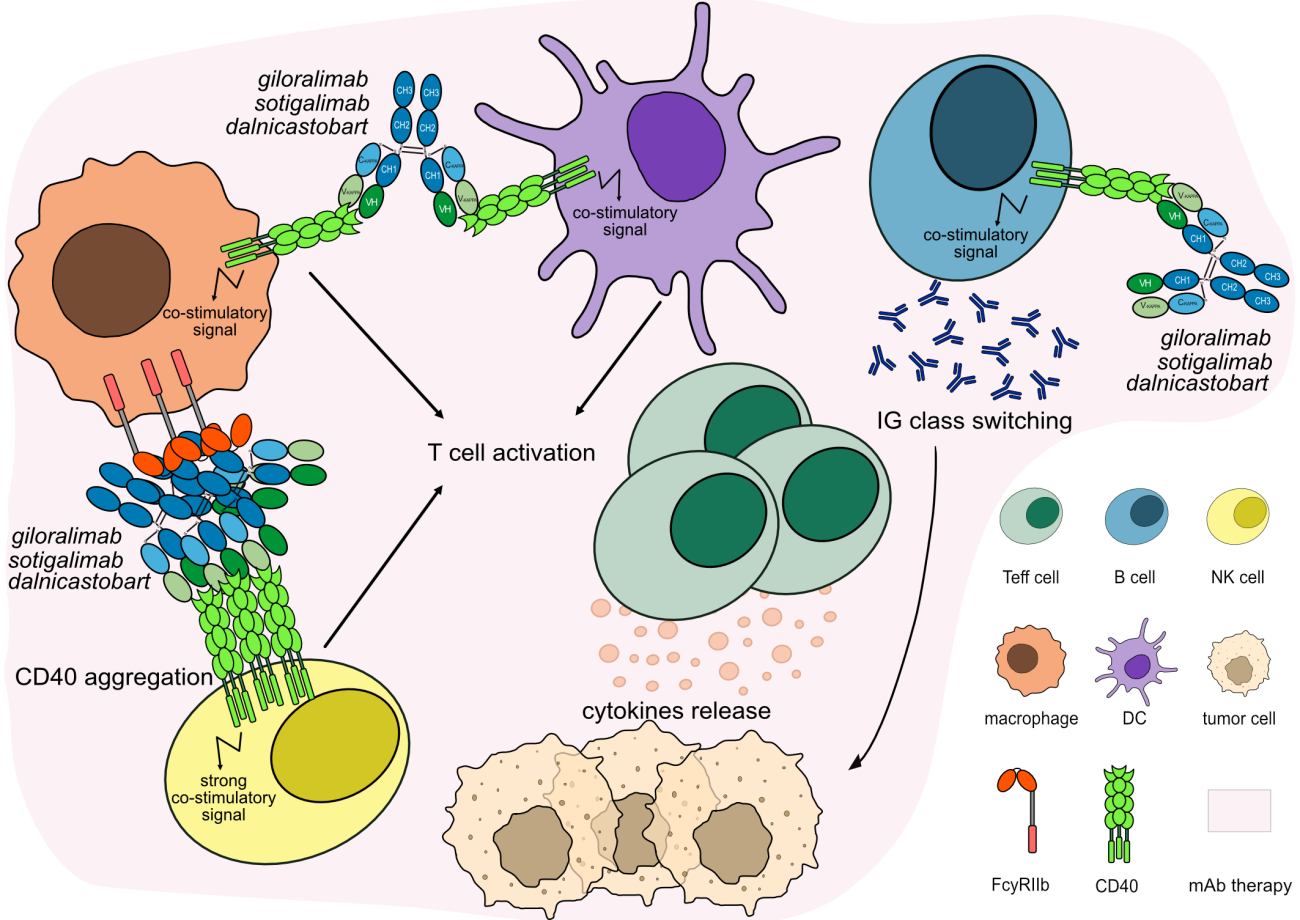
Mechanism of action of agonist mAbs targeting CD40. The antibodies stimulate CD40 signaling to activate and proliferate the immune target cells, which activate IG class switching in B cells and enhance CTL-mediated anti-tumor immune response against tumor cells. The Fc-IgG1 of the antibodies have been engineered to enhance FcγRIIb binding promoting antibody crosslinking and strong CD40 signaling while reducing the binding with FcγRIIIa and inhibiting ADCC activity on APCs. Mechanism of action: Agonist. Effect: Immunostimulant, FcγR crosslinking. (mAb IDs 1012, 1088, 1367).

On the other hand, agonist IgG1 mAbs targeting CD40 with Fc engineered to enhance FcγRIIb binding may mediate antibody crosslinking and strong CD40 signaling while reducing the binding with FcγRIIIa in order to inhibit ADCC activity on APCs expressing CD40 (83, 84) (**Figure 7**). As FcγRIIb is mainly expressed by B cells in tumor tissues, the antibodies are expected to be more active in the TME, depending on the FcγR crosslinking and with lower toxicity. The MOA of these antibodies is ‘Agonist - Immunostimulant, FcγR crosslinking’.

In contrast to the agonist mAbs, antagonist anti-CD40 mAbs block the CD40/CD40LG pathway to inhibit the proliferation of malignant B cells, which highly express CD40, such as in CLL and NHL. In IMGT/mAb-DB, we find one blocking mAb anti-CD40, lucatumumab (mAbID 176), that mediates ADCC and ADCP against tumor cells (85). Thus, lucatumumab’s MOA is ‘Blocking - Fc-effector function’. However, this antibody was discontinued in 2013 after being explored for the treatment of multiple myeloma and follicular lymphoma with modest efficacy as monotherapy (86).

## Discussion

Since the immune system has the potential to recognize and destroy tumor cells, improving immune effector mechanisms against tumors has revolutionized the treatment of several types of cancer (2, 3). Monoclonal antibodies have been utilized to increase the efficiency of anti-tumor T cell responses by precisely targeting immunological checkpoints. Several immune checkpoint targets, co-stimulatory and co-inhibitory receptors that modulate T cell activities, have been discovered to enhance cancer immunotherapy (87–89). This paper describes the MOA of mAbs that target six of the main ICs. mAbs targeting CTLA4, PDCD1, CD274, and LAG3 have been approved by the FDA for second- and first-line treatment against cancer, whereas mAbs targeting ICOS and CD40 are under investigation. Several mAbs targeting the same ICs are being developed, highlighting the interest in IC therapies. These antibodies have different pharmacological properties, specificity, and affinity against the target. It is known that antibodies with high affinity and specificity against the target may enhance clinical outcomes significantly (90).

The description of the mAbs’ MOA allowed to establish two new concepts in the IMGT-ONTOLOGY and define two main mechanisms of action for the studied mAbs, ‘Blocking’ and ‘Agonist’, with similar ‘Immunostimulant’ effects, increasing T cell cytotoxic activity against cancer cells. In cancer immunotherapy, co-inhibitory receptors are blocked by mAbs to restore immune function. The Fc region of mAbs plays an important role in the anti-tumor activity by its effector properties, which can enhance or limit its function, to deplete T_reg_ cells or prevent T_eff_ cells depletion.

Indeed, despite the relevance of the variable region in antigen specific recognition and its binding affinity, the choice of the constant region of a mAb has been shown to play a key role in the effectiveness of the treatment in clinical trials (91). Enhancing the mAb’s ability to bind to FcγRs may increase the anti-tumor activity. Anti-CTLA4 mAbs with enhanced Fc function have been designed to deplete T_reg_ cells, inhibiting its immunosuppressive properties against immune cells in the TME. This depletion enhances anti-tumor immune response in cancer treatment (21). Thus, Fc-enhanced anti-CTLA4 antibodies show anti-tumor activity due to its ‘Fc-effector function’ on T_reg_ cells (92). The term ‘Fc-effector function’ has been added in IMGT to allow querying mAbs where the Fc region plays a role in their MOA.

mAbs blocking the PDCD1/CD274 checkpoints inhibit the co-inhibitory signal on T_eff_ cells and promote cytotoxic activity against tumor cells. The blocking mAbs anti-PDCD1 are mostly IgG4 subclass with limited FcγR binding or IgG1 Fc-silenced to minimize T cells depletion and reduce adverse events. Antibodies with abolished affinity to FcγRs show great anti-tumor efficacy (36) and attempts have been made to develop Fc-silenced mAbs with a better clinical profile than the one provided by an unmuted Fc. Anti-PDCD1 antibodies only have the ‘Immunostimulant’ effect by activating T_eff_ cells, whereas anti-CD274 mAbs with a functional Fc mediated ADCC directly against tumor cells improve tumor killing without unwanted toxicities (93). In addition to ‘Immunostimulant’ effect, anti-CD274 mAbs have the ‘Fc-effector function’ effect in their MOA.

Agonist mAbs against co-stimulatory receptors bind FcγRs signaling immune effector cells against the target cells and allow antibody crosslinking and strong agonism at low levels of the target receptor (52). The mAbs can either bind to FcγRIIIa to increase antibody crosslinking and ADCC activity with ‘Fc-effector function’ effect or preferentially bind to FcγRIIb to mediate antibody crosslinking with limited ADCC activity, an effect defined as ‘FcγR crosslinking’ in IMGT®.

Chain composition, clinical indication, molecular target, and mechanisms of action, may be used to define monoclonal antibodies in IMGT/mAb-DB. Furthermore, amino acid sequences and 3D structures are documented in IMGT/2Dstructure-DB and IMGT/3Dstructure-DB (8), respectively, with reciprocal links to IMGT/mAb-DB. The detailed analysis of the amino acid chains allows the identification of genes and alleles and the delimitation of the antibody regions. The crystal structures of antigen/antibody can provide information for discovering binding modes of antigen/antibody interactions and understanding anti-tumor mechanisms (94, 95).

Thus, IMGT® provides a comprehensive understanding of how monoclonal antibodies work in cancer. An animated video of MOA of FDA-approved therapeutic antibodies targeting ICs, studied in this work, is available here. With the goal of covering the complete IMGT/mAb-DB, IMGT® continues standardized efforts to characterize the mechanisms of action of mAbs targeting various immune checkpoints as well as other targets in cancer treatment and other therapeutic domains, such as autoimmune diseases.

## Data availability statement

The original contributions presented in the study are included in the **Supplementary Material**. Further inquiries can be directed to the corresponding author.

## Author contributions

TM conceived, analyzed the data, and drafted the manuscript. AK and PD analyzed the data and developed the databases. NA and VG discussed and drafted the manuscript. SK conceived and supervised the findings and the write up of this work. All authors contributed to the article and approved the submitted version.

## Glossary

**Table:**

3D	three-dimensional
AA	amino acid
ADCC	antibody-dependent cellular cytotoxicity
ADCP	antibody-dependent cellular phagocytosis
APC	antigen-presenting cell
CD274/PD-L1	programmed death ligand 1
CD40LG	CD40 ligand
CDC	complement-dependent cytotoxicity
CLEC4G/LSECtin	C-type lectin domain family 4 member G
CLL	chronic lymphocytic leukemia
CTL	cytotoxic T lymphocyte
CTLA4	cytotoxic T-lymphocyte associated protein 4
DCs	dendritic cells
EMA	european medicines agency
Fc	fragment crystallizable
FcγRs	Fc-gamma receptors
FDA	U.S. food and drug administration
FGL1	fibrinogen-like protein 1
HGNC	HUGO gene nomenclature committee
ICI	immune checkpoint inhibitor
ICOS	inducible T cell co-stimulator
ICOSLG/CD275	inducible T cell co-stimulator ligand
ICs	Immune checkpoints
IL2	interleukin 2
IL4	interleukin 4
IL5	interleukin 5
IL6	interleukin 6
IL10	interleukin 10
IL12	interleukin 12
INN	international nonproprietary names
LAG3/CD223	lymphocyte activation gene-3
LGALS3/Gal-3	galectin-3
mAb	monoclonal antibody
MH2	major histocompatibility class II
MOA	mechanism of action
NCI	national cancer institute
NHL	non-Hodgkin’s lymphoma
NK	natural killer cell
PDCD1/PD-1	programmed death receptor 1
PDCD1LG2/PD-L2/CD273	programmed death ligand 2
T_eff_	effector T cells
TGFBR2	transforming growth factor beta receptor 2
TME	tumor micro-environment
TNFR	tumor necrosis factor receptor
TNFRSF5	tumor necrosis factor receptor superfamily member 5
TR	T cell receptor
T_reg_	regulatory T cells
WHO	world health organization.
