# Supplementary material for: Mechanisms of action of monoclonal antibodies in oncology integrated in IMGT/mAb-DB
Source: Front Immunol. 2023 May 5;14:1129323. doi: 10.3389/fimmu.2023.1129323 (PMC10196129; doi:10.3389/fimmu.2023.1129323)
Supplement: Supplementary file 1 [file Table_1.docx]

Supplementary Material

Mechanisms of action of monoclonal antibodies in oncology integrated in IMGT/mAb-DB

**Taciana Manso, Anjana Kushwaha, Nika Abdollahi, Patrice Duroux, Véronique Giudicelli, Sofia Kossida***

***Correspondence:** Sofia Kossida (sofia.kossida@igh.cnrs.fr)

**Supplementary Table 1.** Monoclonal antibodies targeting immune checkpoints for cancer immunotherapy with Fc engineered variants according IMGT engineered variant nomenclature. AA changes are defined by the letter of the novel AA and the IMGT position in the domain according IMGT numbering system. The Fc engineered variants are involved in ADCC, CDC, half-life increase, half-IG exchange and glycosylation. Correspondence with the EU numbering are shown. (<https://www.imgt.org/IMGTbiotechnology/> > Antibody glycosylation and effector properties > IMGT engineered variant nomenclature: IGHG variants). With permission from IMGT®, the international ImMunoGeneTics information system®, <http://www.imgt.org>).

| **Target and mAb INN names** | **IMGT engineered Fc variant name** | **IMGT engineered variant definition** | **Correspondence with Eu numbering** | **Effect modification** |
| --- | --- | --- | --- | --- |
| CD274: garivulimab | G1v4 | CH2 A114 | P329A | ADCC reduction  CDC reduction |
| CTLA4: porustobart | G1v7 | CH2 D3, E117 | S239D, I332E | ADCC enhancement |
| CTLA4: botensilimab | G1v8 | CH2 D3, L115, E117 | S239D, A330L, I332E | ADCC enhancement |
| PDCD1: budigalimab, prolgolimab | G1v14 | CH2 A1.3, A1.2 | L234A, L235A | ADCC and CDC reduction |
| PDCD1: penpulimab | G1v14-1 | CH2 A1.3, A1.2, A1 | L234A, L235A, G237A | ADCC and CDC reduction |
| CD274: atezolizumab, betifisolimab  PDCD1: zeluvalimab  LAG3: tuparstobart | G1v29 | CH2 A84.4 | N297A | ADCC reduction  (no *N*-glycosylation site) |
| CD40: sotigalimab | G1v35 | CH2 E29 | S267E | ADCC reduction  Increases FcγRIIb binding |
| CD274: envafolimab | G1v37 | h S5 | C220S | No disulfide bridge inter H-L |
| CD274: durvalumab | G1v39 | CH2 F1.3, E1.2, S116 | L234F, L235E, P331S | ADCC and CDC reduction |
| PDCD1: lodapolimab | G1v43-60 | CH2 A1.3, E1.2, A1, S115, S116 | L234A, L235E, G237A, A330S, P331S | ADCC and CDC reduction |
| CD40: dalnicastobart | G1v47 | CH2 delG1.1 | G236del | ADCC reduction |
| CD274: envafolimab | G1v71 | CH2 A27, G116 | D265A, P331G | ADCC and CDC reduction |
| CD40: giloralimab | G1v72 | CH2 E37 | V273E | ADCC reduction  Increases FcγRIIb binding |
| ICOS: feladilimab | G4v3 | CH2 E1.2 | L235E | ADCC reduction |
| CD274: adebrelimab | G4v4 | CH2 A1.3, A1.2 | F234A, L235A | ADCC and CDC reduction |
| CD274: adebrelimab, pacmilimab, sugemalimab  PDCD1: acrixolimab, balstilimab, camrelizumab, cemiplimab, cetrelimab, dostarlimab, ezabenlimab, geptanolimab, iparomlimab, nivolumab, nofazinlimab, pembrolizumab, pimivalimab, pradusinstobart, pucotenlimab, retifanlimab, sasanlimab, serplulimab, sintilimab, spartalizumab, tislelizumab, toripalimab, zimberelimab  LAG3: encelimab, favezelimab, fianlimab, ieramilimab, miptenalimab, relatlimab  ICOS: feladilimab | G4v5 | h P10 | S228P | Half-IG exchange reduction |
| PDCD1: tislelizumab | G4v6 | CH3 K88 | R409K | Half-IG exchange reduction |
| PDCD1: pucotenlimab | G4v22 | CH2 T16, P91, CH3 A114 | S254T, V308P, N434A | Half-life increase |
| PDCD1: tislelizumab | G4v37 | CH2 P1.4, V1.3, A1.2, A27, V92 | E233P, F234V, L235A, D265A, L309V | ADCC and CDC reduction |
| LAG3: fianlimab | G4v38 | CH2 P1.4, V1.3, A1.2, delG1.1 | E233P, F234V, L235A, G236del | Reduces FcγR binding |

Supplementary Table 2. Blocking anti-PDCD1 mAbs present in IMGT/mAb-DB and their mechanisms of action (MOA).

| **INN mAbs** | **Isotype** | **IMGT variant (Fc-silenced)** | **IMGT MOA** | **Clinical trials** |
| --- | --- | --- | --- | --- |
| [acrixolimab](https://www.imgt.org/mAb-DB/mAbcard?AbId=1236#moa_Oncology) | IgG4 | - | **Blocking**  Immunostimulant | Phase I/II  (NCT04450901) |
| [balstilimab](https://www.imgt.org/mAb-DB/mAbcard?AbId=912#moa_Oncology) | IgG4 | - |  | Phase II  (NCT05632328) |
| [budigalimab](https://www.imgt.org/mAb-DB/mAbcard?AbId=845#moa_Oncology) | IgG1 | [G1v14](https://www.imgt.org/IMGTbiotechnology/IGHG_variant/Tableau1.html) |  | Phase II  (NCT04807972) |
| [camrelizumab](https://www.imgt.org/mAb-DB/mAbcard?AbId=659#moa_Oncology) | IgG4 | - |  | Phase III  (NCT04639180, NCT04928807, NCT05097209, NCT05313282) |
| [cemiplimab](https://www.imgt.org/mAb-DB/mAbcard?AbId=846#moa_Oncology) | IgG4 | - |  | Phase M  (first approval in 2018) |
| [cetrelimab](https://www.imgt.org/mAb-DB/mAbcard?AbId=809#moa_Oncology) | IgG4 | - |  | Phase III  (NCT04658862) |
| [dostarlimab](https://www.imgt.org/mAb-DB/mAbcard?AbId=849#moa_Oncology) | IgG4 | - |  | Phase M  (first approval in 2021) |
| [ezabenlimab](https://www.imgt.org/mAb-DB/mAbcard?AbId=1009#moa_Oncology) | IgG4 | - |  | Phase II  (NCT04719988) |
| [finotonlimab](https://www.imgt.org/mAb-DB/mAbcard?AbId=1128#moa_Oncology) | IgG4 | - |  | Phase III  (NCT04171284) |
| [geptanolimab](https://www.imgt.org/mAb-DB/mAbcard?AbId=1077#moa_Oncology) | IgG4 | - |  | Phase II  (NCT03623581) |
| [iparomlimab](https://www.imgt.org/mAb-DB/mAbcard?AbId=1203#moa_Oncology)* | IgG4 | - |  | Phase III  (NCT05690945) |
| [lodapolimab](https://www.imgt.org/mAb-DB/mAbcard?AbId=955#moa_Oncology) | IgG1 | [G1v43-60](https://www.imgt.org/IMGTbiotechnology/IGHG_variant/Tableau1.html) |  | Phase I |
| [nivolumab](https://www.imgt.org/mAb-DB/mAbcard?AbId=424#moa_Oncology) | IgG4 | - |  | Phase M  (first approval in 2014) |
| [nofazinlimab](https://www.imgt.org/mAb-DB/mAbcard?AbId=1211#moa_Oncology) | IgG4 | - |  | Phase III  (NCT04194775) |
| [pembrolizumab](https://www.imgt.org/mAb-DB/mAbcard?AbId=472#moa_Oncology) | IgG4 | - |  | Phase M  (first approval in 2014) |
| [penpulimab](https://www.imgt.org/mAb-DB/mAbcard?AbId=1093#moa_Oncology) | IgG1 | [G1v14-1](https://www.imgt.org/IMGTbiotechnology/IGHG_variant/Tableau1.html) |  | Phase III  (NCT04974398, NCT05244642) |
| [pimivalimab](https://www.imgt.org/mAb-DB/mAbcard?AbId=1082#moa_Oncology) | IgG4 | - |  | Phase I/II  (NCT04669899) |
| [pradusinstobart](https://www.imgt.org/mAb-DB/mAbcard?AbId=1365#moa_Oncology)* | IgG4 | - |  | Phase I  (NCT05075993) |
| [prolgolimab](https://www.imgt.org/mAb-DB/mAbcard?AbId=856#moa_Oncology) | IgG1 | [G1v14](https://www.imgt.org/IMGTbiotechnology/IGHG_variant/Tableau1.html) |  | Phase III  (NCT03912389, NCT03912415) |
| [pucotenlimab](https://www.imgt.org/mAb-DB/mAbcard?AbId=1142#moa_Oncology) | IgG4 | [G4v22](https://www.imgt.org/IMGTbiotechnology/IGHG_variant/Tableau1.html) |  | Phase III  (NCT05652894, NCT05647954, NCT04486651) |
| [retifanlimab](https://www.imgt.org/mAb-DB/mAbcard?AbId=968#moa_Oncology) | IgG4 | - |  | Phase II  (NCT04116073) |
| [sasanlimab](https://www.imgt.org/mAb-DB/mAbcard?AbId=969#moa_Oncology) | IgG4 | - |  | Phase III  (NCT04165317) |
| [serplulimab](https://www.imgt.org/mAb-DB/mAbcard?AbId=970#moa_Oncology) | IgG4 | - |  | Phase III  (NCT05353257) |
| [sintilimab](https://www.imgt.org/mAb-DB/mAbcard?AbId=859#moa_Oncology) | IgG4 | - |  | Phase III  (NCT04840290, NCT03748134) |
| [spartalizumab](https://www.imgt.org/mAb-DB/mAbcard?AbId=761#moa_Oncology) | IgG4 | - |  | Phase III  (NCT04229004) |
| [tislelizumab](https://www.imgt.org/mAb-DB/mAbcard?AbId=757#moa_Oncology) | IgG4 | [G4v37](https://www.imgt.org/IMGTbiotechnology/IGHG_variant/Tableau1.html) |  | Phase III  (NCT03358875) |
| [toripalimab](https://www.imgt.org/mAb-DB/mAbcard?AbId=863#moa_Oncology) | IgG4 | - |  | Phase III  (NCT04376866) |
| [zeluvalimab](https://www.imgt.org/mAb-DB/mAbcard?AbId=1153#moa_Oncology) | IgG1 | [G1v29](https://www.imgt.org/IMGTbiotechnology/IGHG_variant/Tableau1.html) |  | Phase I/II  (NCT04185883) |
| [zimberelimab](https://www.imgt.org/mAb-DB/mAbcard?AbId=1071#moa_Oncology) | IgG4 | - |  | Phase III  (NCT04736173) |
| [pidilizumab](https://www.imgt.org/mAb-DB/mAbcard?AbId=453#moa_Oncology) | IgG1 | - | **Blocking**  Immunostimulant  Fc-effector function | Phase I/II  (NCT05480449) |
| [lipustobart](https://www.imgt.org/mAb-DB/mAbcard?AbId=1373)** | IgG4 | - | - | Phase I  (NCT03286296) |
| [rulonilimab](https://www.imgt.org/mAb-DB/mAbcard?AbId=1179)** | IgG1 | - | - | Phase II/III  (NCT05408221) |

*Monoclonal antibodies with a MOA suggested by IMGT® owing to a lack of scholarly papers giving proof of their pre-clinical effects. Their MOA may evolve as new data emerge. IMGT® suggestion is based on i) the function of the mAb target in the cancerous environment and ii) the analysis of their Fc region, when possible.

** No information from the literature to describe the MOA.
